# Single-cell lipidomics: performance evaluation across four liquid chromatography mass spectrometry (LC-MS) systems

**DOI:** 10.1039/d5an00851d

**Published:** 2025-09-23

**Authors:** Johanna von Gerichten, Kyle D. G. Saunders, Matt Spick, Melanie J. Bailey

**Affiliations:** a School of Chemistry and Chemical Engineering, Faculty of Engineering and Physical Sciences, University of Surrey Guildford UK; b School of Health Sciences, Faculty of Health and Medical Sciences, University of Surrey Guildford UK; c Department of Infectious Diseases, Guy's Hospital, King's College London London UK melanie.j.bailey@kcl.ac.uk

## Abstract

Single-cell lipidomics holds tremendous promise for understanding a wide range of pathological conditions involving heterogenous cell populations, including infection, cancer, diabetes, and cardiovascular disease and yet its widespread adoption has been hitherto limited. Although Liquid Chromatography Mass Spectrometry (LC-MS) is a globally established method for lipidomics, its application to single cells has been considered particularly challenging, if not impossible, due to the very low sample volume and the high dynamic range and structural complexity of cellular lipids. Recent advances have shown that LC-MS-based single-cell lipidomics is achievable, offering the benefit of sampling cells in their native state, as well as chromatographic separation to reduce matrix effects and enhance peak annotation. In this study, we advocate for wider adoption of single-cell lipidomics by demonstrating that a range of widely accessible LC-MS platforms can successfully generate single-cell lipid profiles. Using four distinct instrumental configurations, we provide a perspective on the achievable depth of coverage and annotation. We show that polarity switching, ion mobility spectrometry, and electron-activated dissociation significantly enhance both lipidome coverage and confidence in lipid identification from single cells.

## Abstract 2

Single-cell lipidomics promises to transform our understanding of diseases. We show by that a range of widely accessible LC-MS platforms can successfully generate single-cell lipid profiles.

## Graphical abstract

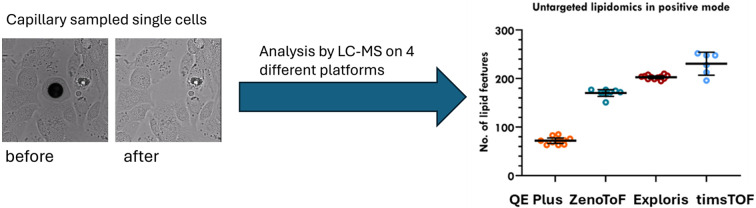

## Introduction

Lipidomics, the comprehensive analysis of lipids within biological systems, has emerged as a powerful approach for understanding disease pathology and cellular function. Dysregulated lipid profiles have been implicated in a broad range of conditions, including cancer, neurodegenerative disorders, and infectious diseases.^1–3^ Most lipidomics studies have traditionally relied on bulk sample analysis using liquid chromatography-mass spectrometry (LC-MS), owing to the enormous structural diversity and concentration span of lipids across biological samples. However, such population-averaged approaches can obscure crucial insights into cellular heterogeneity, especially when lipids play pivotal roles in energy storage, membrane integrity, and intracellular signaling.^4^

To decipher lipid-mediated processes at the level of individual cells, particularly in contexts such as microenvironmental interactions or bystander effects, single-cell lipidomics has become increasingly desirable.^5,6^ Indeed, the broader field of single-cell omics has gained significant traction as the functional significance of subcellular and cell-specific mechanisms in biology has been recognized.^7,8^ To this end, various platforms are under active development to isolate and analyse single cells, including mass spectrometry imaging (MSI), microfluidics, and capillary-based techniques.^9^ These technologies have uncovered cell type specific lipid signatures across various tissues, *e.g.* prostate,^6^ kidney,^10^ liver^11^ and pancreas^12^ and contributed to the formulation of the “lipotype” hypothesis.^13^ Notably, compositional lipid differences in fibroblasts at different skin depths,^14^ monocyte-to-macrophage differentiation,^10^ and foam cell formation^15^ illustrate the biological relevance of lipidomic profiling at single-cell resolution. Similar approaches have yielded insights into neutrophil activation during infection,^16^ hepatocyte response to high-fat diet in non-alcoholic fatty liver disease models,^11^ and drug resistance in colorectal cancer cells.^17^

Most studies so far have employed high-spatial-resolution mass spectrometry imaging techniques such as matrix assisted laser desorption/ionisation (MALDI),^10^ secondary ion mass spectrometry (SIMS)^18^ or nano desorption electrospray ionisation (DESI).^19^ However a limitation of these approaches is that cells are not analysed in their native state, and in the case of MALDI, require application of a matrix and normally require introduction into vacuum chamber, which may perturb analytes. Additionally, because MSI techniques lack chromatographic separation, they are susceptible to matrix effects, leading to reduced quantification capabilities and annotation confidence.^20^ In contrast, single cell isolation techniques such as capillary sampling and microfluidics enable the collection of live cells and can retain spatial context, while allowing compatibility with LC-MS.^21–25^ While microfluidics enables higher throughput, capillary sampling captures user-selected cells under microscope observation, and thus can be used to select specific cell typies in a co-culture, or cells displaying specific morphological or fluorescent markers, whilst also preserving environmental and positional fidelity. Recent work by Kontiza *et al.* has shown that both sampling strategies yield comparable lipidomic profiles.^25^ Nevertheless, many studies still favour direct infusion *via* nanospray, foregoing the advantages of chromatographic separation.^6,15,26^

Therefore despite growing interest, LC-MS-based single-cell lipidomics is still underrepresented in the literature^12,25,27,28^ and important opportunities remain to enhance its sensitivity, selectivity, and throughput. Recent advances in instrumentation now permit greater sensitivity through enhanced ion transmission efficiency and improved selectivity through methods such as polarity switching, ion mobility spectrometry, and electron-activated dissociation (EAD).

In this study, we explore the feasibility and scope of single-cell lipidomics using LC-MS across four different instrument configurations, including both high-end and more widely accessible platforms of ion mobility, polarity switching and EAD fragmentation in the context of single cell lipidomics. Whilst this approach can be used with a variety of cell types, we chose to use a cell line to avoid introducing heterogeneity that would confuse methodological differences. Using capillary-selected single human pancreatic adenocarcinoma cells, we demonstrate untargeted single cell lipid profiling can be achieved using analytical to nano flow rates, and highlight the capabilities. Our findings reveal both overlapping and complementary capabilities across these systems. This provides a resource to enable broader adoption of LC-MS-based single-cell lipidomics in biomedical research.

## Methods

### Cell culture

Human pancreatic adenocarcinoma cells PANC-1 (Merck, UK) were cultured in DMEM glucose (Sigma-Aldrich, UK, cat no. 21969035) with 10% (v/v) fetal bovine serum (Fisher Scientific, UK, cat no. 11550356), 1% penicillin/streptomycin (Fisher Scientific, UK, cat no. 15140122), and 2 mM l-glutamine (Sigma-Aldrich, UK, cat no. 25030024). Cells were kept at 37 °C with 21% O_2_ and 5% CO_2_. Cell culture media was replaced on alternate days and cells were passaged approximately once a week when confluency reached 80%. 48 hours prior to single cell sampling, 200 000 cells were seeded into a 3.5 cm Nunc™ Glass Bottom Dishes (150682 Thermo, UK). 2 mL of cell culture media (no cells) was aliquoted into a cell culture dish to serve as negative control. Cells were washed twice with 37 °C FBS-free culture medium and left in 2 mL FBS-free culture medium for cell sampling.

### Capillary single cell sampling

Cells were washed with warm FBS-free media before capillary sampling and kept in fresh FBS-free media. The 35 mm culture dish was introduced to the Yokogawa SS2000 Single Cellome System™, where living cells were sampled individually into 10 μm capillaries (Yokogawa). Cells were kept at 37 °C with 5% CO_2_ during sampling. Cells were manually selected at random with following pressures: pre-sampling 6 kPa, sampling 14 kPa and post-sampling 3 kPa. The cells were sampled with a single pick and held for 200 ms. The capillary tips were immediately frozen on dry ice after cell sampling.

Cells were transferred from the sampling capillaries into QSert LC-MS vials (Waters) by backfilling the capillaries with 5 μL lysis solvent that consisted of 51 : 62 : 87 IPA/H_2_O/ACN spiked with EquiSPLASH (Avanti polar lipids, cat no. 330731; 16 ng mL^−1^), and using a gas syringe with a Luer lock adapter to elute the solution into the vial, as described previously.^23^ Cells for the nano-flow workflows were freeze-dried in a FreeZone 2.5 L bench top freeze dryer (Labcono, USA) under low vacuum of 0.5 mbar and stored under nitrogen. Freeze-dried cells were then shipped from the United Kingdom to either Thermo Fisher-California or Bruker-Germany and stored at −80 °C.

To make representative blank samples (taking into account a small volume of cell media that may be aspirated along with the cell), we aliquoted 2 mL of cell culture media (no cells) into a cell culture dish and kept it in the incubator alongside the cells. The cell media was than diluted in the starting mobile phase to a blank concentration of 1 nL cell media per uL.

### LC-MS platforms

In this work we tested the feasibility of carrying out single cell lipidomics on three high end LC-MS systems, and a fourth older generation system widely available in many labs worldwide. The systems tested varied in the columns, flow rates, mass spectrometry acquisition and the compatible data analysis software, but can be broadly characterised as (1) analytical flow with MS1 acquisition only (no MS/MS), (2) microflow with MS2 spectra collected using electron activated dissociation, (3) nanoflow with polarity switching and MS2 (4) nanoflow with ion mobility and MS2. For simplicity, the methods are referred to by the mass spectrometry platform that was used. Details of the LC gradients and data analysis parameters used can be found in the SI.

### Q Exactive Plus method (analytical flow and MS1)

Cells were analysed using a Thermo Fisher Scientific (Massachusetts, USA) Ultimate 3000 UHPLC system coupled to a Thermo Fisher Scientific Q-Exactive Plus Orbitrap mass spectrometer. The ionisation source was a heated electrospray ionisation (HESI) probe set to 320 °C, automatic gain control (AGC) was active with a target of 1 × 10^6^, HESI probe spray voltage of 4 kV, and mass range *m*/*z* 200–1400 was acquired with a resolution of 140 000 as described previously.^23^

### ZenoToF method (microflow and DDA)

Cells were analysed using an Acquity M-class (Waters) coupled to a 7600 ZenoTOF system (Sciex) as described previously.^12^ Positive ESI parameters were: 4500 V spray voltage, 80 V declustering potential, 350 °C source temperature; *m*/*z* range 150–900 with MS^1^ resolution of 44 000 and MS^2^ resolution of 42 000 at *m*/*z* 760.5, collision energy MS^1^ 12 V and CID 35 V. The top 30 fragment ions were detected with 0.2 ms acquisition time and without dynamic background correction.

### Exploris method (nanoflow and polarity switching)

The analysis was conducted on a Vanquish Neo UHPLC (Thermo Fisher, USA) coupled to an Orbitrap Exploris™ 240 (Thermo Fisher, USA). Data was acquired with MS^1^ resolution of 60 000 and an *m*/*z* window of 250–1250. Data-dependent MS^2^ was performed at 15 000 resolution, taking the top 4 scans and HCD set to 35 V. Polarity switching was used to obtain both positive and negative spectra, switching between polarities every 10 scans. On the day of analysis, LC-MS vials were removed from −80 °C storage and samples were reconstituted with 7 μL IPA before being vortexed and sonicated. 7 μL H_2_O was added to each vial and 12 μL per sample was injected onto the column.

### timsTOF method (nano-flow, ion mobility and MS^2^)

Samples were analysed using a Bruker timsTOF Ultra with a default 4D Lipidomics PASEF method: mass range 100–1350 *m*/*z* with MS^1^ resolution and MS^2^ resolution of 60 000 at *m*/*z* 1222, mobility range 0.55–1.90 1/*K*_0_, ramp time 100 ms. Nano-LC was performed using a Bruker nanoElute. Samples were redissolved in 2.3 μL (butanol/IPA/H_2_O 8 : 23 : 69) and 2 μL were injected (*n* = 6). Mobile phase blank and FBS free cell media blank were redissolved in 50 μL (butanol/IPA/H_2_O 8 : 23 : 69) and 2 μL were injected (for each three technical replicates).

## Results

### Characterising the lipidome of single pancreatic cancer cells using different LC-MS platforms

A simple method optimisation was performed on each instrument to maximise the coverage of low volume lipids using a cell extract, as reported previously in Von Gerichten *et al.*^12^ We then analysed the response of the internal standard (EquiSPLASH) using each system. As shown in Fig. S1, the coefficient of variation (%CV) for EquiSPLASH analytes increases from 10% under ideal conditions (direct addition of standard to LC-MS vial) to 15% after transfer from the sampling capillaries to LC-MS vials (Fig. S1B; A_MS1 and M_MS2), to 30% after transfer and then shipping (Fig. S1B; N_MS2_PS and N_MS2_IMS).

**Fig. 1:**
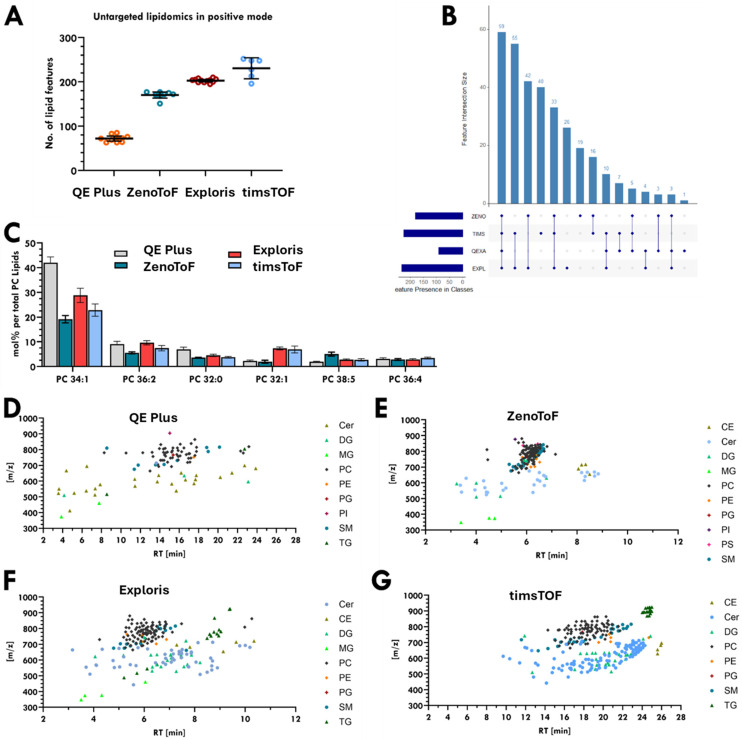
Lipid coverage in positive ion mode for single pancreatic cancer cells from different LC-MS(/MS) platforms. (A) Average number of lipid features detected in single cells. Q Exactive Plus method with analytical flow LC; *n* = 10 single cells, ZenoTOF DDA method with microflow LC; *n* = 8 single cells, Exploris 240 method with nanoflow LC, DDA and polarity switching; *n* = 12 single cells, timsTOF Ultra method with nanoflow LC and PASEF using ion mobility; *n* = 6 single cells. (B) UpSet plot visualising overlap of identified lipid features across four LC-MS(MS) platforms. (C) Top 6 most abundant PC's in single PANC-1 cells. (D–G) Retention time *vs.* different *m*/*z* values detected by each platform, coloured by assigned lipid class.

Fig. 1A shows the average number of lipid features detected in positive mode in single cells for each method. The presented lipids are restricted to the 9 lipid classes detected in the internal standard EquiSPLASH (PC, PE, PS, PI, PG, DG, TG, SM, Cer) and were processed with a 50% detection rate filter. This was done to normalise the lipid signals to the internal standards and have a checkpoint for the retention times. The average number of detected lipids in one single cell increases from analytical flow (72 ± 11%) to micro-flow (170 ± 5%), with the highest numbers of lipids detected for the nano-flow methods (203 ± 2% and 231 ± 10%). This comparison is based on positive mode data but for the first nano-flow method, the data was acquired only with polarity switching, which might impact total number of scans as the instrument was not run in optimal positive mode conditions.

Fig. S2 illustrates a t-SNE plot of lipids detected in single cells across all four analytical methods, demonstrating clear separation, consistent with their methodological differences. Chromatograms of analytes from the internal standards (Fig. S1C) show that the same lipid classes from the internal standard are detected by all four methods, although with some notable differences in lipid class separation. Despite these variations, Fig. 1B and C reveal areas of consistency. The UpSet plot in Fig. 1B shows that 99% of lipids identified in single cells *via* the QE Plus method are also detected by at least one other method. The ZenoToF and timsToF techniques share 79–88% of identified lipids, while 12–21% of lipids are uniquely detected by each method (Fig. S3). Fig. 1C presents the average signal intensities of the six most abundant phosphatidylcholine (PC) species in single PANC-1 cells. Although variations in mol% (particularly from the QE Plus method) are evident due to differences in lipid coverage, the trends and dynamic ranges are broadly consistent across all single-cell datasets.

Fig. 1D–G shows the distribution of the different lipid classes over the LC gradient for the lipids detected in the single cells. The relatively short 15-minute gradient for ZenoToF and Exploris methods (Fig. 1E and F) results in most analytes eluting in a very small time window (at around 6 minutes), in contrast to the longer (30 minutes) QE Plus and the timsToF method (Fig. 1D and G), where compounds elute over a longer time window. Overall, these plots illustrate the differences in sensitivity and selectivity to single-cell lipids, rising from the analytical flow (Fig. 1D) to micro- (Fig. 1E) to nano-flow (Fig. 1F and G) observable through the increased density of the datapoints as well as a higher variety of colours for each datapoint. The effect of the gradient length is also visualised by the clearer clusters for each lipid class comparing a short (Fig. 1F) and long (Fig. 1G) nano-flow gradient.

### Polarity switching in single cell lipidomics increases coverage and assignment confidence

**Fig. 2:**
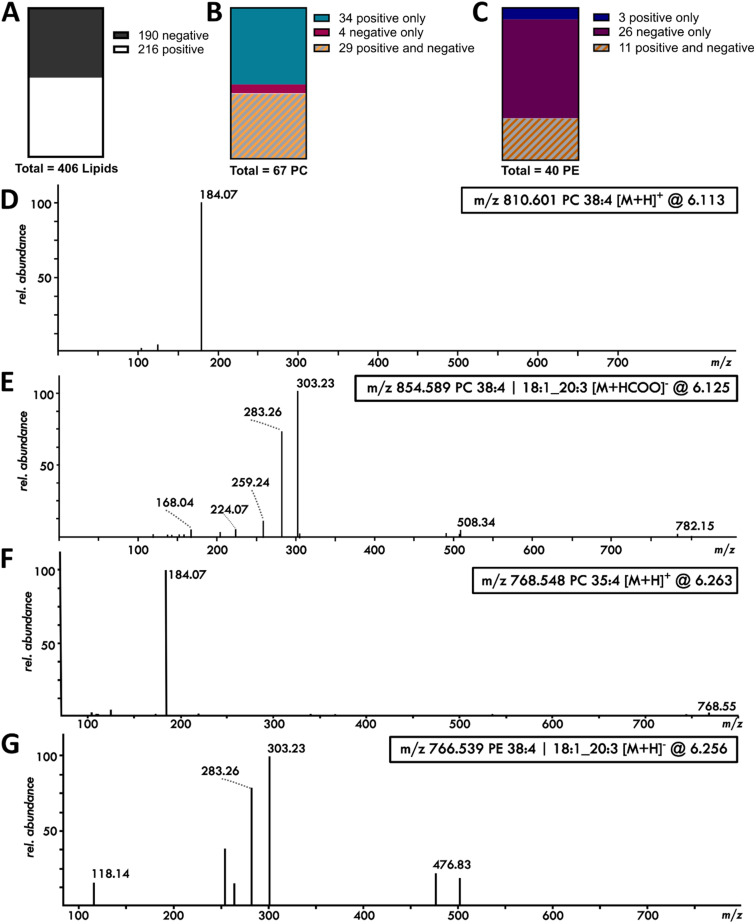
Polarity switching for single cell lipidomics based on the Exploris method, using nano-flow and DDA/MS2. (A) Number of lipids detected in a single cell in positive and negative mode based on EquiSPLASH. (B) Number of detected PCs in positive mode only and in positive and negative mode. (C) Number of detected PEs in a single cell in positive mode only and in positive and negative mode. (D) and (E) Positive and negative mode fragmentation mass spectrum of PC (38:4) from a single cell, demonstrating the increased information for chain length based on negative mode (PC 18:1_20:3). (F) Positive mode fragmentation spectrum of PC 35:4 (peak at 6.26 minutes) from a single cell and (G) negative mode fragmentation spectrum of the same peak identifying it as PE 38:4.

Polarity switching allows the detection of positive as well as negative compounds within the same chromatography peak, reducing the time available for the mass spectrometer to detect masses but increasing the information gained. Applying polarity switching to single pancreatic cancer cells resulted in the detection of 406 lipids in the Exploris method, of which 190 lipids were detected in negative mode, therefore increasing the lipid coverage from the positive mode by 47% (Fig. 2A). A number of lipids were detected in positive as well as negative mode, which increased confidence in identification, with additional fatty-acyl chain information from the negative fragmentation spectrum. For example, of the total 67 PCs identified, 29 PCs were detected in positive as well as negative mode (Fig. 2B). The positive ion fragmentation mass spectrum of PCs in single cells mainly shows the PC headgroup fragment at *m*/*z* 184.07 and barely any additional fragments, as shown in Fig. 2D for PC (38:4). However, PC (38:4) is also detected as a [M + HCOO]^−^ adduct in negative mode, with clear negative ion fragmentation for the fatty-acyl chains 18:1 (*m*/*z* 283.26) and 20:3 (*m*/*z* 303.23) as well as the fatty acid loss of 20:3 (*m*/*z* 508.34) shown for a single cell in Fig. 2E.

Another example is the phosphatidylethanolamines (PE). Only 14 PE and etherPE are identified in positive mode, but an additional 26 PE and etherPE can be detected in negative mode in single cells (Fig. 2C). The two modes are highly complementary – for example, PC (35:4) is identified in positive mode within a peak at 6.26 minutes, as shown by the PC headgroup fragment of *m*/*z* 184.07 in Fig. 2F. The negative [M + HCOO]^−^ adduct of PC (35:4) is not detected in single cells, meaning positive mode is required for this analyte as the sensitivity in negative mode is not enough to detect this compound. Conversely, PE (38:4) is identified in negative mode in the same peak at 6.26 minutes, as shown by the two acyl chain fragments 18:1 (*m*/*z* 283.26) and 20:3 (*m*/*z* 303.23) in Fig. 2G and the additional fragments of *m*/*z* 196.04 and *m*/*z* 480.31 in a bulk extract diluted to the level of 7 cells per injection (Fig. S4). PE (38:4), however, is not detected in positive mode due to a too low abundance as positive ion.

Overall this is a demonstration of the increase in detection power when using polarity switching for LC-MS to enhance confidence in identification of lipids in a single cell dataset. The same statement also applies for etherPC, PG and PI as shown in Fig. S5.

### Ion mobility spectrometry in single cell lipidomics for the separation of isobars and isomers to increase assignment confidence and coverage

**Fig. 3:**
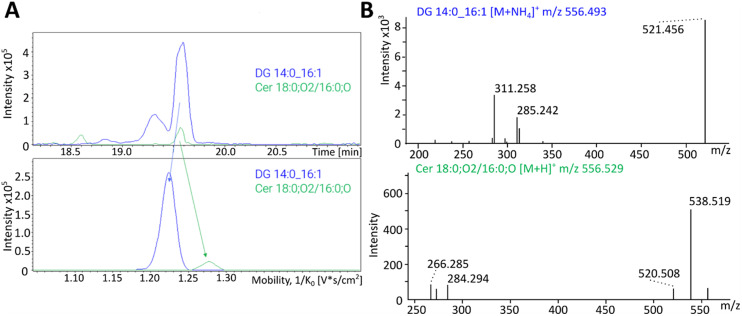
Representative isobar separation with ion mobility from a single PANC-1 cell using nano-flow LC (nanoELute) coupled to a timsTOF Ultra. (A) Top: Co-eluting peaks at 19.6 minutes for DG (14:0_16:1) (blue) and Cer (18:0;O2/16:0;O) (green). Bottom: Separated peaks *via* ion mobility for DG (14:0_16:1) (blue; CCS 1.22) and Cer (18:0;O2/16:0;O) (green; CCS 1.28). (B) Top: MS^2^ fragment spectrum for DG (14:0_16:1) with major masses for the acyl chain fragments (14:0 *m*/*z* 311.258 and 16:1 *m*/*z* 285.242) and backbone fragment (*m*/*z* 521.456). Bottom: MS^2^ fragment spectrum for Cer (18:0;O2/16:0;O) with major masses for the 18:0;O2 sphingolipid backbone and corresponding water loss (*m*/*z* 284.294 and 266.285, respectively) as well as the water loss and double water loss of the molecular ion (*m*/*z* 538.52 and 520.51, respectively).

Ion mobility spectrometry (IMS) acts as a fourth dimension to the analytical capacity of lipidomics in addition to the common dimensions of liquid chromatography (retention time), exact mass (MS^1^) and fragmentation-based identification (MS^2^). There are different techniques and instrumental set-ups commercially available, but the basic principle is that of ions separated in gas phase according to their mobility or size per charge, which is expressed as calculation of cross-section (CCS).^29,30^ Applied to lipids in bulk analysis, IMS has been shown to separate isobars that co-elute on LC columns, most commonly PC and PE, and even separate lipid isomers that for example differ in *cis* and *trans* double bonds.^31,32^ However, Fig. 3 shows an example of lipid isobars in a single cell with diacylglycerol DG (14:0_16:1) and ceramide Cer (18:0;O2/16:0;O) that co-elute at 19.6 minutes on the LC (Fig. 3A top) but are separated by IMS with a different CCS of 1.22 and 1.28 (Fig. 3A bottom) before entering the mass detector. Fig. 3B shows the fragmentation spectra of these compounds in a single cell after IMS, reflecting a separation with clear fragments for DG as well as Cer. As the ceramide has a much lower intensity than the DG, it is very possible that the lipid annotation software would not have picked up on the ceramide fragments overlayed by the DG fragments without the separation. One of the greatest challenges in single cell lipidomics is to detect and accurately annotate low abundant lipids within a cell. As shown here, IMS has the possibility to do this by removing background signals and separating isomers. However, the IMS separation does not only create cleaner MSMS spectra, as the acquired CCS values provides also an additional annotation level and increase the confidence level of annotations by matching against predicted values or values from libraires.

### Electron-activated dissociation of lipids in single cells for detailed structure identification

**Fig. 4:**
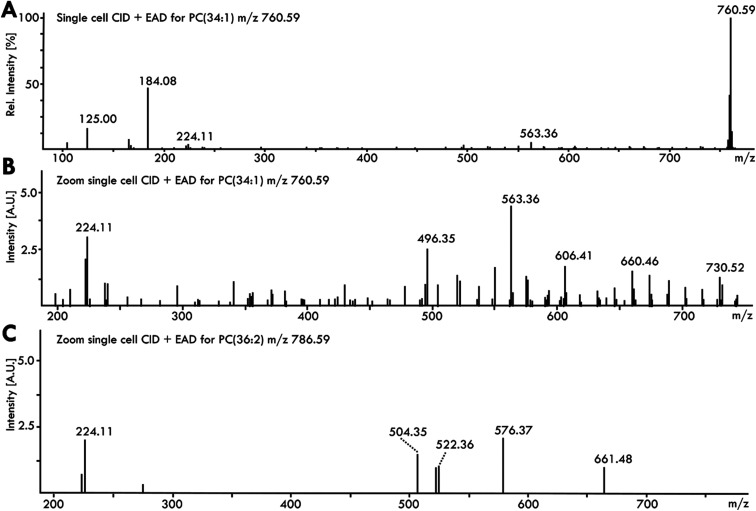
Fragmentation of phospholipids from a single pancreatic cancer cell using EAD. (A) PC (34:1) mass spectrum using CID and EAD for *m*/*z* 760.59. (B) Zoomed in fragmentation spectrum using CID followed by EAD fragmentation of PC (34:1) with highlighted key fragments. (C) Zoomed in fragmentation spectrum using CID followed by EAD fragmentation of PC (36:2).

Both collision-induced dissociation (CID) for MS^2^ and IMS can only provide limited information on fatty acyl chain *sn*-position, double bond position and regioisomerism of double bonds on the acyl chain. For a detailed structure elucidation, electron-activated dissociation (EAD) techniques such as electron impact excitation of ions from organics (EIEIO) can be used for fragmentation. EIEIO has been shown to distinguish lipid regioisomers, chain length position and *sn*-position, double bond position and even differences between *cis* and *trans* double bonds. Using a combination of CID/EAD in a targeted approach (MRM^HR^) for PC (34:1) and PC (36:2) in single pancreatic cancer cells, fragments beyond the prominent *m*/*z* 184.07 are generated in positive ion mode (Fig. 4A). The peaks between *m*/*z* 730.52 and *m*/*z* 632.43 show the Δ14 pattern of the C18:1 acyl chain (C_*n*_H_2*n*_ + 2 fragments) with the break in delta between *m*/*z* 660.46 and *m*/*z* 606.41, indicating the double bond position at Ω9 (Fig. 4B). The prominent ketene fragment ion loss at *m*/*z* 496.35 suggests the C18:1 is at the *sn*-2 position,^33,34^ resulting in a more detailed structure of PC (34:1) as PC (16:0_18:1(9)) in single pancreatic cancer cells. The EAD fragments were identified with the MSDial EAD library and confirmed with a PANC-1 bulk cell sample equivalent to 7 injected cells as well as Avanti Porcine Brain Polar Lipid standard (Fig. S6). Even for PC (36:2), a PC roughly 25% the abundance of PC (34:1) in single PANC-1 cells (see Fig. 1D), there is enough information from the CID/EAD fragmentation spectrum to identify it as PC (18):1(9)/18:1(9) (Fig. 4C). Fragments at *m*/*z* 520.36 and *m*/*z* 522.36 indicate the loss of acyl chain and fragments *m*/*z* 661.48 and *m*/*z* 576.37 reflect the shift to the C_*n*_H_2*n*_ − 1 and C_*n*_H_2*n*_ loss after the double bond at position Ω9.

Overall, these examples show that it is very well possible to perform structure identification work in a single cell with EAD, which in the future could be a major source of informing discovery style untargeted lipidomics work.

## Discussion

This work underscores the growing feasibility and accessibility of single-cell lipidomics *via* LC-MS, facilitated by advances in capillary sampling and microfluidics for cell isolation. As LC-MS platforms are already widespread in analytical laboratories, we demonstrate that single-cell lipidomics is not limited to specialized instrumentation but can be deployed across a range of commonly available systems.

Accurate structural elucidation of lipids is especially critical given that minor differences in headgroup, acyl chain length, or unsaturation can markedly influence biological function. We have shown here that recent advances in MS instrumentation, such as enhanced acquisition speeds, data-dependent fragmentation, alternative fragmentation modes and ion mobility modules offer considerable advantages in this context. For single-cell lipidomics, faster scan rates improve sensitivity to low-abundance species and enable more comprehensive fragmentation, thereby enhancing structural resolution.

Although our analysis focused on a single cell type, processed within a single laboratory using consistent sampling and transfer protocols, a major challenge remains – the absence of universally accepted reference materials for single-cell analysis. Even in bulk lipidomics, method comparisons across instruments are hampered by differences in acquisition modes, ion sources, and data processing pipelines, as previously reported in inter-platform benchmarking studies.^35–37^ These factors introduce systematic biases that complicate direct comparisons. This limitation also applies to this work, but this does not undermine the ability to highlight the broad applicability and complementary nature of single cell lipdomics approaches.

In this study, we applied stringent data filtering (specifically that the signals had to be present in 50% of cells, unlike single cell proteomics, where frequency filters are not typically applied) to mitigate the effects of cell-to-cell heterogeneity and focus on reproducibly detected lipid features. We also restricted our analysis to lipid classes covered by our internal standard mixture, which necessarily limits total number of species detected, but enhances identification confidence. Despite this conservative approach, the analytical-flow system reliably detected ∼100 annotated lipids per cell, with the lower flow rate platforms yielding even broader coverage. These levels are comparable to lipid detection in bulk extract analyses^12^ (Fig. S6), and the comparability of results across all four LC-MS(/MS) configurations indicate that robust, untargeted single-cell lipidomics is achievable using capillary sampling with chromatographic separation.

Future studies could expand the detected lipidome by incorporating more comprehensive internal standards to cover more lipid classes, or relaxing detection thresholds. Broader lipid coverage, however, must be balanced against the risk of introducing false positives or reducing confidence in annotations.^38^ Additionally, efforts to increase LC throughput and optimize MS acquisition modes will be key to enabling high-content, high-throughput single-cell lipidomics. Further, the establishment of community-wide reference standards and quality control procedures is essential for benchmarking and cross-platform reproducibility.

## Conclusions

This study demonstrates the feasibility, adaptability, and complementary strengths of available LC-MS(/MS) platforms for performing single-cell lipidomics. By evaluating diverse instrument configurations across independent laboratories, we establish that single-cell lipidome profiling is both technically achievable and broadly accessible. We have shown how analytical strategies such as polarity switching, ion mobility spectrometry, and EAD significantly enhance lipidome coverage and structural resolution, even when applied to single cells.

Noting the difficulties in providing a robust comparison between instruments, we can draw some comparisons between the overall methods used. The greatest lipid coverage of 406 lipids per single cell was achieved using the Orbitrap Exploris method, with MS2 acquisition in a run time of just 16 minutes, making it a very attractive option for single cell lipidomics. Whilst the ZenoToF and timsTOF platforms demonstrated high confidence lipid annotation, this came at the cost of coverage, and in the case of the timsToF method, increased run time. Therefore users of single cell lipidomics should consider whether the goal of their experiment is to detect as many lipids as possible, or to have very high confidence in their annotation, because at present these cannot be achieved in a single method.

Our findings offer a practical framework for researchers seeking to implement or optimize single-cell lipidomics using existing infrastructure. Moreover, they highlight future directions for method development, including integration with high-throughput sampling, novel separation methods to improve throughput and sensitivity, data harmonization, and refined structural annotation workflows. As single-cell approaches continue to reshape molecular biology, LC-MS-based lipidomics stands poised to uncover previously hidden dimensions of cellular heterogeneity with direct relevance to disease, development, and therapeutic response.

## Author contributions

MB conceived and designed the study. JG and KS carried out the experiments and, together with MS analysed the data. JG wrote the first draft of the article with input from all authors. All authors contributed to the article and approved the submitted version.

## Conflicts of interest

The authors declare no competing financial interest.

## Supplementary Material

AN-150-D5AN00851D-s001

## Data Availability

The raw and processed data relating to this article will be made available to researchers on request, by contacting the corresponding author. Supplementary information (SI) is available. See DOI: https://doi.org/10.1039/d5an00851d.
