# Supplementary material for: Single-cell lipidomics: performance evaluation across four liquid chromatography mass spectrometry (LC-MS) systems
Source: Analyst. 2025 Sep 23;150(20):4525–34. doi: 10.1039/d5an00851d (PMC12462284; doi:10.1039/d5an00851d)

Supplementary Information (SI) for Analyst.
This journal is © The Royal Society of Chemistry 2025

Supplementary Information

Liquid Chromatography

QExactive Plus: The gradient started with 70% A for 5 minutes, then rapidly decreased to 57% A within 0.1 min to slowly further decrease to 30% A over 8.9 minutes. The gradient then decreases to 1% A over 7 minutes before holding 1% A for 3 minutes. Finally, the gradient increases to 70% A within 0.1 min and then holding 70% A for 4 minutes.

ZenoToF: The LC gradient decreased from 60 % A at 0.5 min to 1 % A at 4.5 min, stays isocratic for 1.5 min and increases from 20 % A at 6.5 min to 60 % A at 11 min and stays isocratic for 4 min. Data was acquired using Sciex OS (Version 3.0.339).

Exploris The gradient started at 70 % A for 1 minute, decreased to 45 % A for 2 minutes, then decreased further to 25 % A for 2 minutes, decreased again to 5 % A for 4 minutes, decreased to 0 % A for 3 minutes, remained isocratic for 4 minutes, then increased to 70 % A for 0.2 minutes, remaining isocratic for 3.8 minutes.

timsTOF: The gradient starts with 1% B for 1 minute before increasing to 30% B over 2 minutes, followed by increasing to 51% over 4 minutes and then increasing to 61%, 70% and 99% B over 5 minutes each. The gradient stays isocratic for 5 minutes at 99% B before decreasing to 1% B over 1 minute and staying at 1% B for 2 more minutes.

	QE Plus Analytical flow + MS^1	ZenoToF Micro-flow + MS^2	Exploris Nano-flow + MS^2 with polarity switching	timsTOF Nano-flow + IMS + MS^2
Flow rate [µL/min]	350	8	0.75	0.35
Sample solvent	51:62:87 IPA/H2O/ACN	51:62:87 IPA/H2O/ACN	1:1 IPA/H2O	Butanol/IPA/H2O 8:23:69
Injection volume [µL]	15	5	12	2
Column	C30 Accucore (150x2.1mm, 2.6 µm)	Phenomenex Luna Omega Polar C18 (50x0.3mm, 3 µm)	Thermo Scientific EASY-Spray PepMap (75 µm x15 cm)	Aurora C18 1.6 µm (75 µm x 25 cm)
Column temperature [°C]	40	40	45	60
Mobile Phase A	60:40 ACN/H2O			
Mobile Phase B	85:10:5 IPA/Water/ACN	85:10:5 IPA/Water/ACN	88:10:2 IPA/ACN/H2O	90:10 IPA/ACN
Additives	10 mM ammonium formate and 0.1% formic acid			
Run time [min]	30	20	15	30

Data Analysis

MS-Dial (v5.4.241004) was used to process the raw LC-MS/MS data from Thermo Scientific and Sciex. The following parameters were changed from default for data processing:

	Analytical flow + MS^1	Micro-flow + MS^2	Nano-flow + MS^2 with polarity switching
Data collection MS^1 [Da]	0.001	0.01	0.01
Data collection MS^1 [Da]	N/A	0.025	0.025
Amplitude	5,000	300	8,000 pos / 1,000 neg
Identification MS^1 [Da]	0.001	0.01	0.01
Identification MS^2 [Da]	N/A	0.02	0.02
Identification RT tolerance [min]	0.05	0.1	0.2
Alignment RT tolerance [min]	0.1	0.5	0.5
Alignment MS^1 [Da]	0.005	0.01	0.01

Smoothing was carried out using linear weighed moving average with 3 scan smoothing level and 5 scan minimum peak width. Adducts of $[M+H]^+$, $[M+NH4]^+$ and $[M+H-H_2O]^+$ were allowed. Gap filling by compulsion was disabled. A signal-to-background cut-off >3 was applied and values were background-corrected for cell media blanks. Further analysis was carried out in Excel; blank corrected signals were subjected to a 50% detection rate filter applied and signals were normalised to the internal standard. Only the following lipid classes covered by the internal standard are reported: LPC, Cer, MG, DG, PC, PE, PI, PG, PS, SM and TG including the ether-form of these lipids. GraphPad Prism version 8.4.3 for Windows (GraphPad Software, San Diego, California USA) was used for statistics and figures.

timsTOF Nano-flow and MS^2 with ion mobility

Data acquired with the Bruker instrument was processed using MetaboScape (2023b). Spectral library search (MS/MS data) and rule-based lipid annotation was used. The narrow annotation window had the following parameters: *m/z* 2ppm, RT 0.1 min, mSigma 50, MS/MS score 800 and CCS 1%. The wide annotation window had the following parameters: *m/z* 5ppm, RT 0.5 min, mSigma 250, MS/MS score 400 or 200 and CCS 5 or 3%. A Signal-to-background cut-off >3 was applied. An algorithm for RT correction and CCS correction was applied to detect outliers.

Supplementary Figure S 1*: EquiSPLASH internal standard – 9 lipid classes measured with four different platforms; Average internal standard variance in (A) mobile phase (n=9) and (B) single cells as percent covariance (QE Plus n=10, ZenoToF n=8, Exploris n=12, timsTOF n=8); (C) Chromatogram of internal standard classes in a single cell from different platforms in the order QE Plus, ZenoTOF, Exploris, TimsTOF method.*

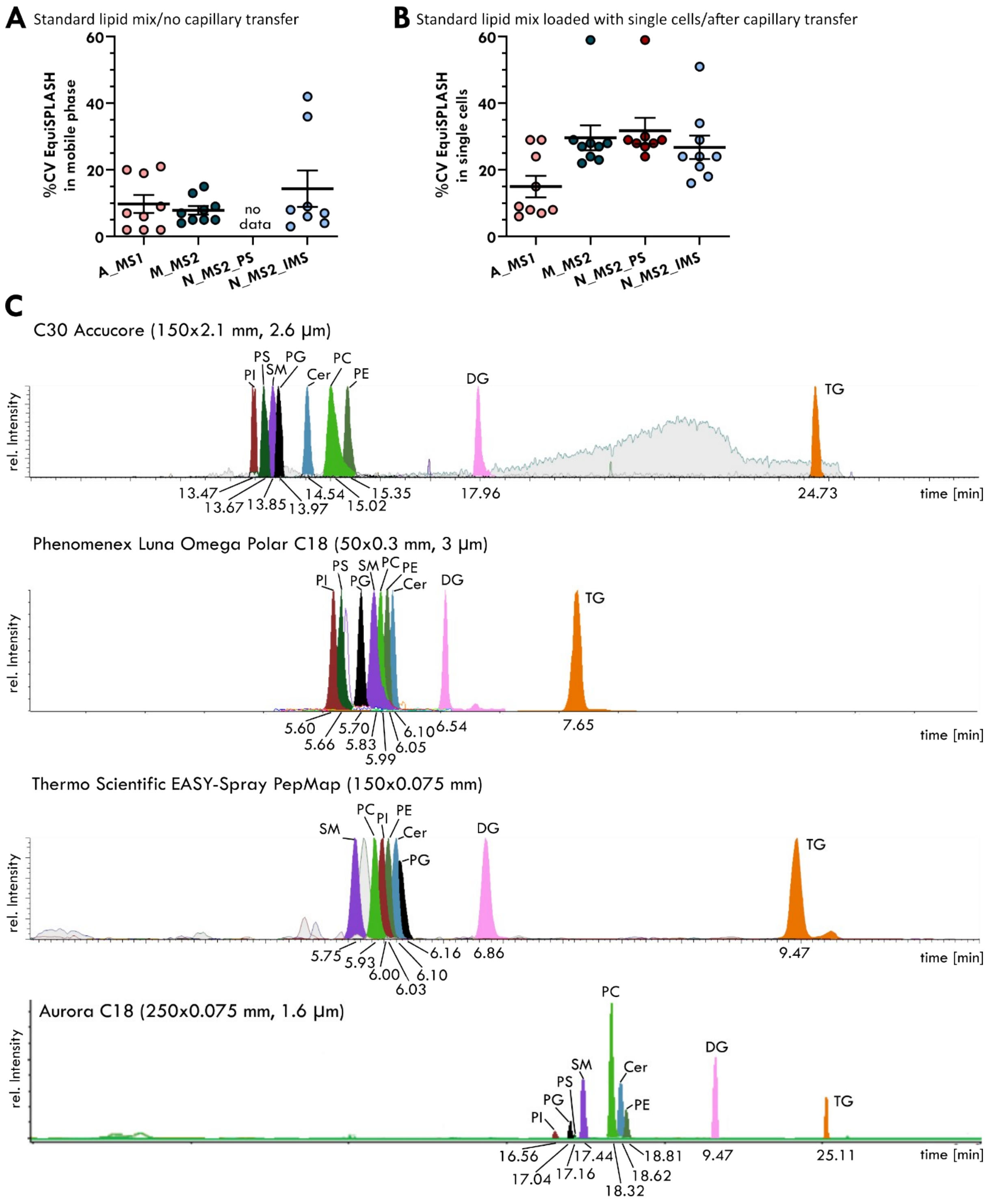

Supplementary Figure S 2*: t-SNE plot comparing lipid features from single PANC-1 cells detected with four different LC-MS(MS) methods. (EXPL=nano-flow with polarity switching on Orbitrap Exploris 240, QEXA= analytical flow with Orbitrap QExactive Plus, TIMS= nano-flow with ion mobility on timsTOF Ultra, ZENO= micro-flow on ZenoTOF).*

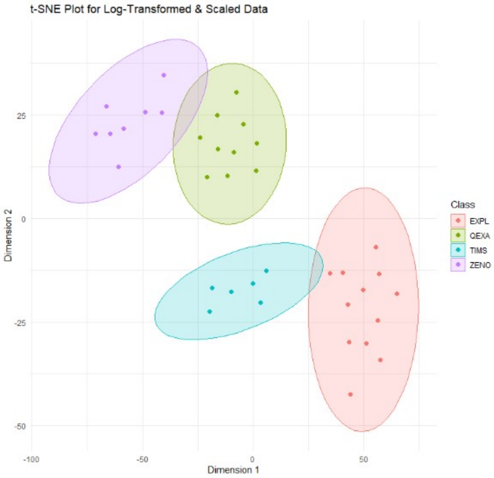

Supplementary Figure S 3*: Venn diagram for three different LC-MS/MS platform: micro-flow ZenoToF, nano-flow Exploris 240 and nano-flow TimsTOF Ultra comparing lipid features in single PANC-1 cells.*

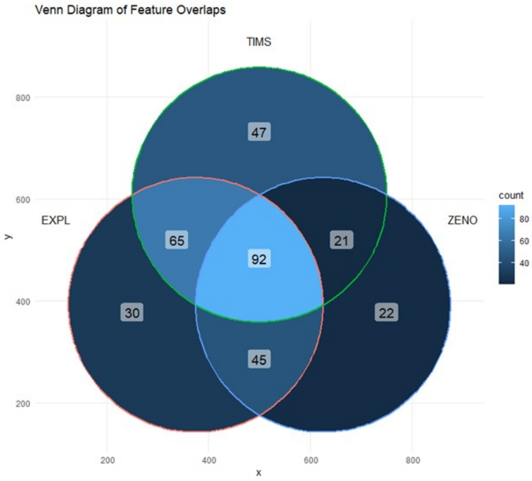

Supplementary Figure S 4: *Fragment spectrum of PE 38:4 m/z 766.54 in negative mode from a bulk cell extract of 7 cells.*

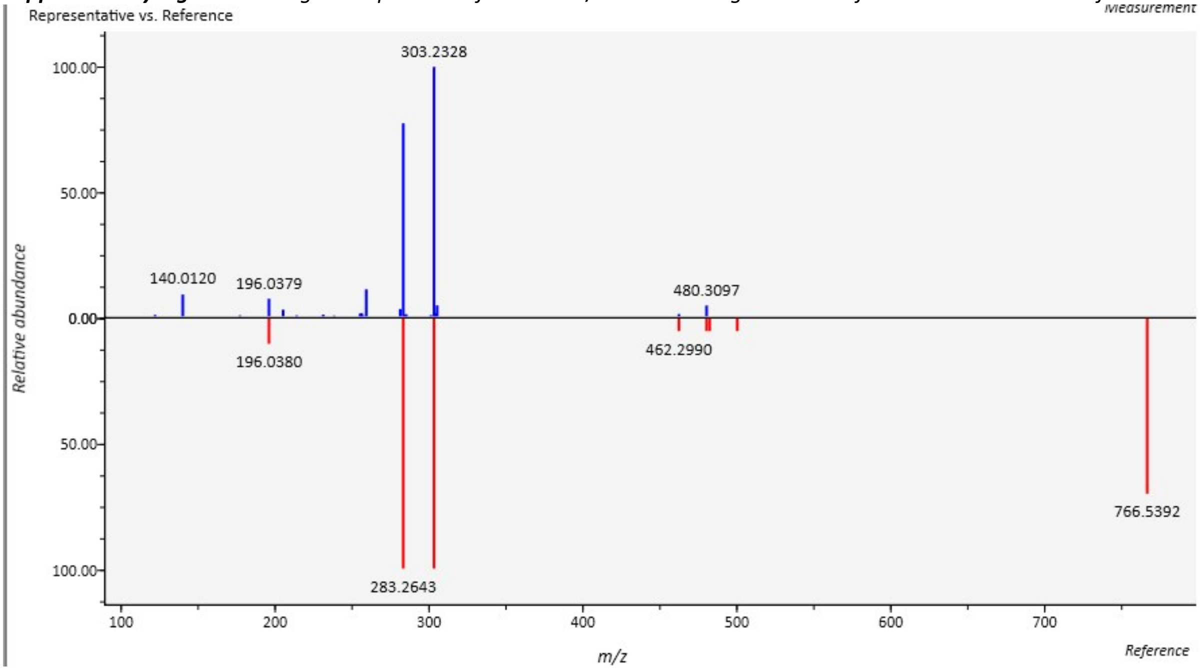

Supplementary Figure S 5*: Positive (left) and negative (right) ion fragmentation spectra of selected lipids in single PANC-1 cells. Spectra are acquired with Vanquish Neo UHPLC coupled to an Orbitrap Exploris™ 240 (Thermo Fisher, USA) using polarity switching.*

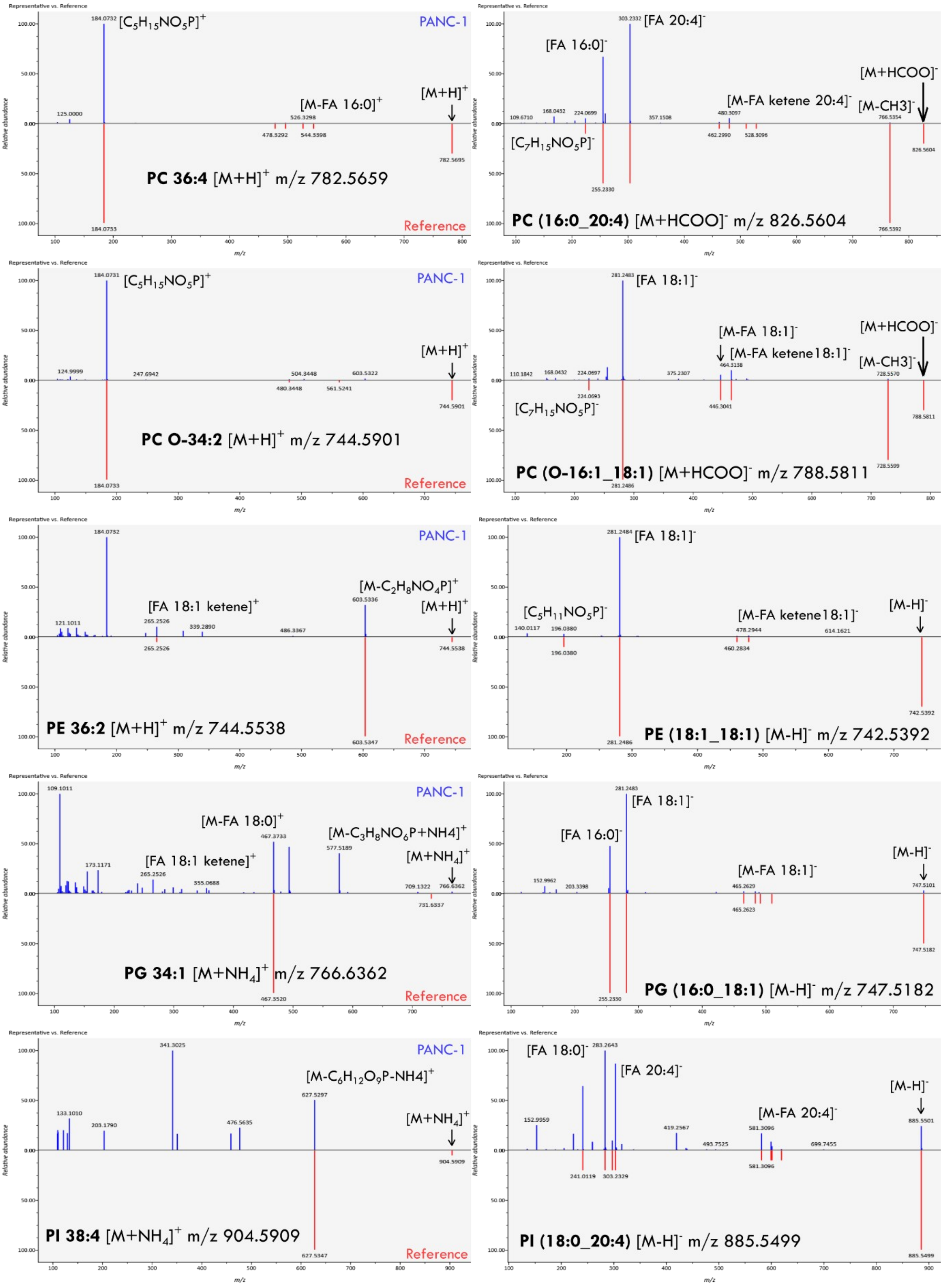

Supplementary Figure S 6 *Fragmentation of PC(34:1) using CID and EAD for m/z 760.59 in single pancreatic cancer cells, bulk cell extract and avanti porcine brain polar lipid extract for m/z 470-690. Highlighting the -18:1(7) acylchain loss at m/z 496.34, the -16:0 acylchain loss at m/z 522.36 and the doublebond fragments with the break in delta 14 between m/z 660.46 and m/z 606.41, indicating the double bond position at Ω9.*

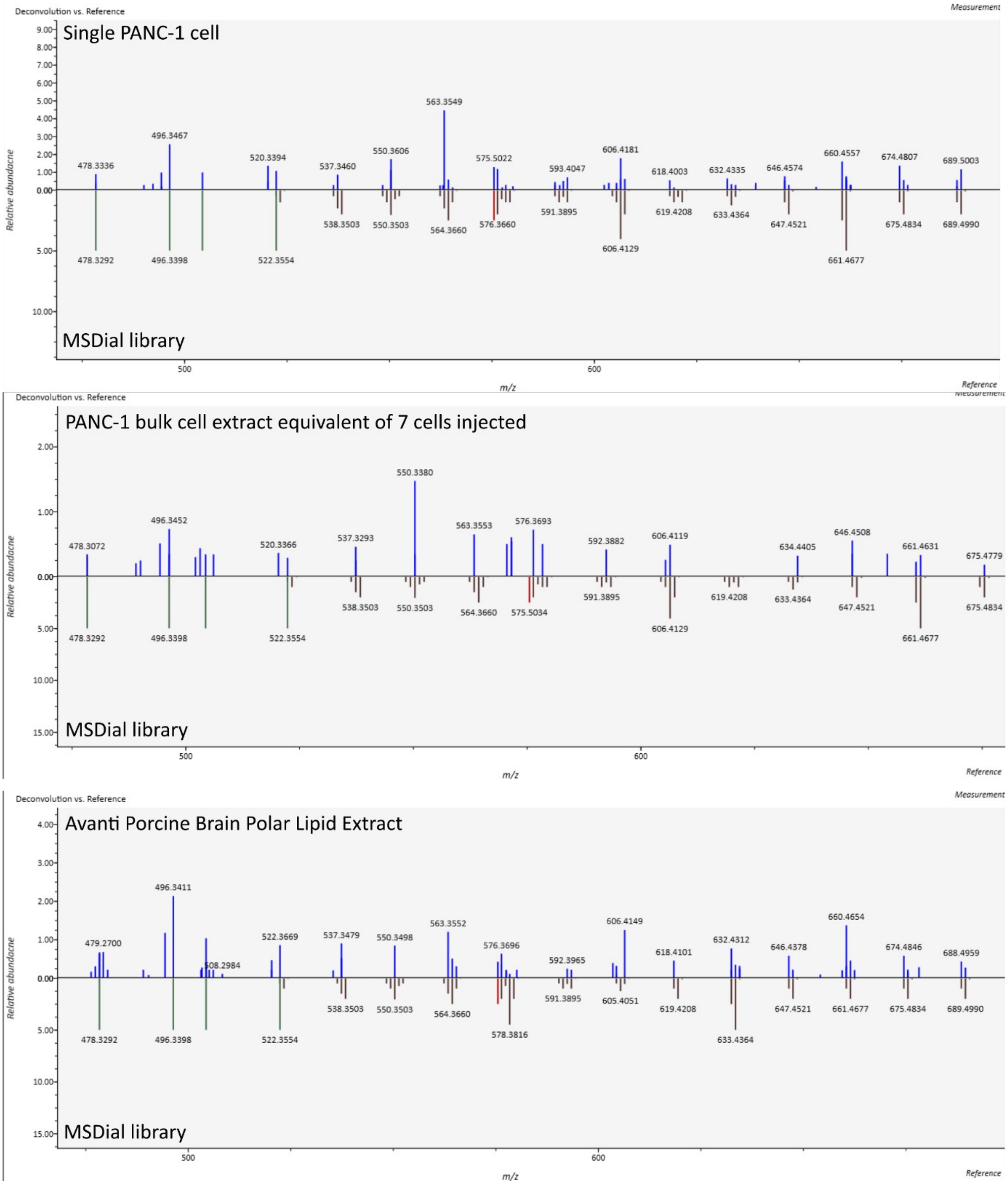

Supplementary Figure S 7: *The number of lipid features in a PANC-1 bulk cell extract as described in von Gerichten et al 2024*[8] *when an equivalent of 14 or 140 cells are injected compared to single cel analysis and the number of lipid features in single PANC-1 cells. Both analysed in the same LC-MS run using nano-flow and a Bruker TimsTOF Ultra as described in methods.*

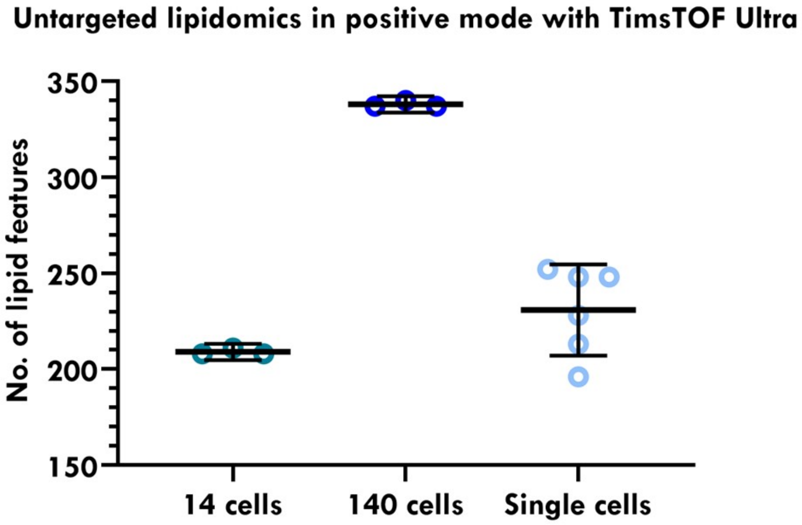

Supplement: AN-150-D5AN00851D-s001 [file AN-150-D5AN00851D-s001.pdf]
